# Roles and Regulation of Extracellular Vesicles in Cardiovascular Mineral Metabolism

**DOI:** 10.3389/fcvm.2018.00187

**Published:** 2018-12-21

**Authors:** Mark C. Blaser, Elena Aikawa

**Affiliations:** ^1^Division of Cardiovascular Medicine, Department of Medicine, Center for Interdisciplinary Cardiovascular Sciences, Brigham and Women's Hospital, Harvard Medical School, Boston, MA, United States; ^2^Division of Cardiovascular Medicine, Department of Medicine, Brigham and Women's Hospital, Center of Excellence in Cardiovascular Biology, Harvard Medical School, Boston, MA, United States

**Keywords:** extracellular vesicles, exosomes, calcification, mineralization, atherosclerosis, calcific aortic valve disease

## Abstract

Cardiovascular calcification is a multifaceted disease that is a leading independent predictor of cardiovascular morbidity and mortality. Recent studies have identified a calcification-prone population of extracellular vesicles as the putative elementary units of vascular microcalcification in diseased heart valves and vessels. Their action is highly context-dependent; extracellular vesicles released by smooth muscle cells, valvular interstitial cells, endothelial cells, and macrophages may promote or inhibit mineralization, depending on the phenotype of their originating cells and/or the extracellular environment to which they are released. In particular, emerging roles for vesicular microRNAs, bioactive lipids, metabolites, and protein cargoes in driving this pro-calcific switch underpin the necessity of innovative strategies to employ next-generation sequencing and omics technologies in order to better understand the pathobiology of these nano-sized entities. Furthermore, a recent body of work has emerged that centers on the novel re-purposing of extracellular vesicles and exosomes as potential therapeutic avenues for cardiovascular calcification. This review aims to highlight the role of extracellular vesicles as constituents of cardiovascular calcification and summarizes recent advances in our understanding of the biophysical nature of vesicle accumulation, aggregation, and mineralization. We also comprehensively discuss the latest evidence that extracellular vesicles act as key mediators and regulators of cell/cell communication, osteoblastic/osteoclastic differentiation, and cell/matrix interactions in cardiovascular tissues. Lastly, we highlight the importance of robust vesicle isolation and characterization when studying these phenomena, and offer a brief primer on working with cardiovascular applications of extracellular vesicles.

## Introduction

**Figure 1:**
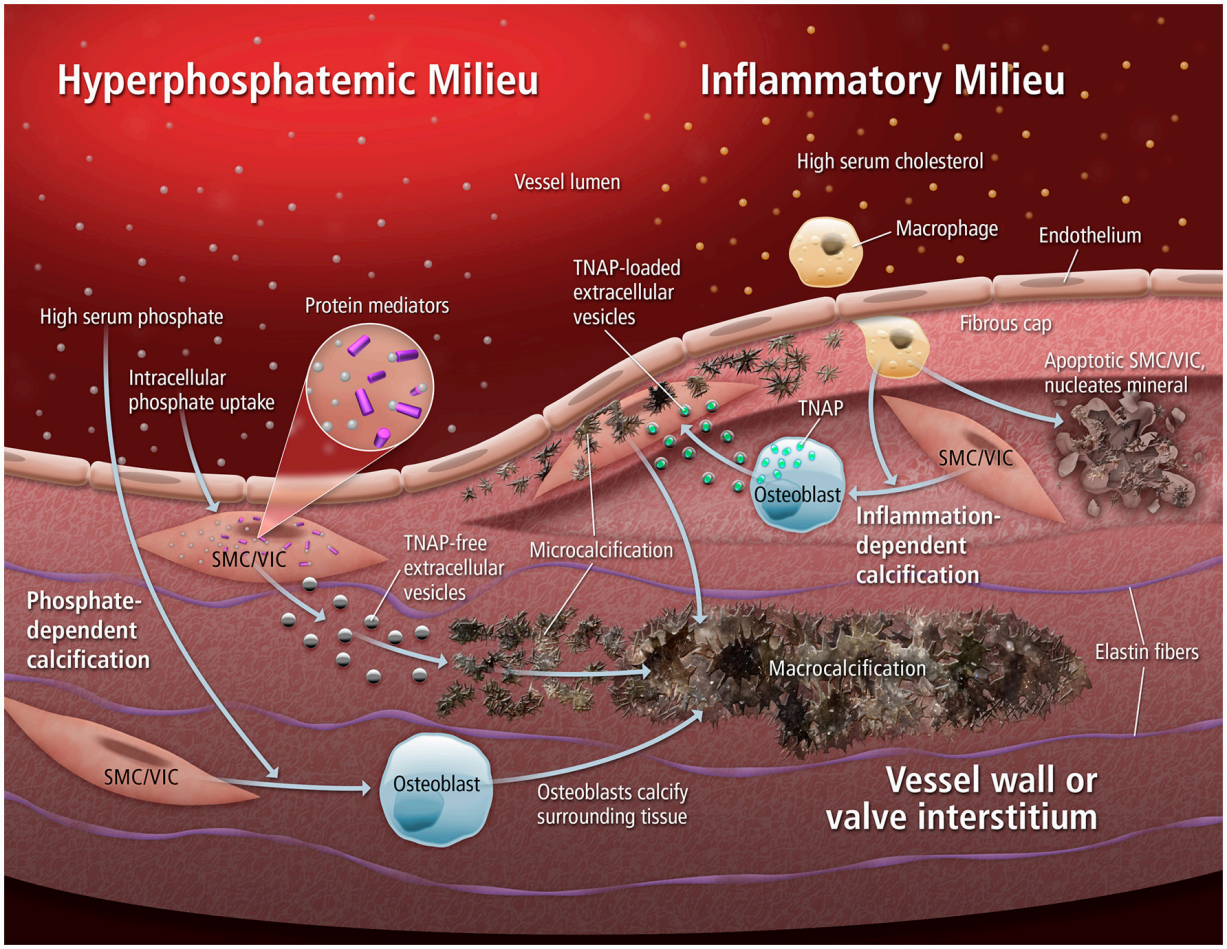
Mechanisms of calcification. Extracellular vesicles can mediate multiple forms of cardiovascular calcification, in both hyperphosphatemic and inflammatory milieus. When an abundance of phosphate is present, rapid tissue calcification can occur through the nucleation of mineral by EVs low in TNAP. Alternatively, inflammatory stimuli can drive osteogenic differentiation of resident SMCs/VICs that leads to the release of TNAP-loaded EVs that progressively aggregate into microcalcifications. From (4).

Cardiovascular calcification is both a strong predictor of morbidity and mortality (1, 2) as well as a direct driver of cardiovascular events (3). In a mineralization-promoting milieu (e.g., atherosclerosis, diabetes, chronic kidney disease (CKD)], such calcification occurs when vascular smooth muscle cells (SMCs) or circulating cells differentiate to an osteogenic-like phenotype that actively synthesizes an extracellular matrix (ECM) and regulates the deposition of hydroxyapatite [Figure 1, reviewed in (5)]. This mineral nucleation often begins as microcalcifications, which can potentiate atherosclerotic plaque rupture by concentrating mechanical forces within the fibrous cap (6, 7). Such calcification is not limited to the vasculature: in the cardiac valves, resident valvular interstitial cells (VICs) undergo transformation into myofibroblast-like and osteoblast-like cells, leading to thickening, stiffening, fibrosis, and calcification of the valve leaflets. In turn, this calcific burden impairs leaflet opening/closure, resulting in stenosis or regurgitation and a deadly load on the cardiac muscle: aortic stenosis conveys a 75% 5-year risk of heart failure, valve replacement surgery, or mortality (8, 9).

Expressed ubiquitously by all human cell types, extracellular vesicles (EVs) are found throughout the body's tissues and biofluids (10). EVs are membrane-bound, nanometer-scale structures that contain cargo (RNA, proteins, and metabolites) and whose secretion is highly conserved between forms of eukaryotic life [reviewed in (11)]. The release of these particles was originally thought to be a mode of waste elimination (12), but they are now understood to play an active role in the regulation of cellular processes. For example, EVs are known to mediate the adaptive immune response (13), modulate tumor metastasis (14), and control coagulation cascades (15). For some time, there has been a growing body of evidence that calcifying EVs released from SMCs are strongly implicated in mineralization of the vasculature (16). Cell types known to be involved in cardiovascular calcification release membrane-bound EVs that have the potential to nucleate minerals, aggregate into microcalcifications, and drive the pathological differentiation of neighboring tissues (17–19). It appears likely then that the release of these calcifying EVs initiates and drives the progression of vascular calcification. Besides taking part in homeostasis and pathogenesis, attempts are being made to exploit the unique properties of these structures [low toxicity, stable phenotypes, targeted delivery, extended half-life; reviewed in (20)] for other purposes. Recent studies by our lab and others have identified many putative anti-calcific and anti-fibrotic proteins that may be amenable to arresting both the origins and advancement of vascular calcification (21–23); therapeutic transport of these or other compounds by EVs could allow for a leap in the treatment of cardiovascular diseases. Treatment efforts directed at vascular/valvular calcification have to date been largely ineffective, due in part to low delivery efficiency and non-specific targeting in the complex multi-cell type calcifying vascular milieu [reviewed in (24)]. An acellular EV treatment approach would reduce regulatory burdens and is amenable to multiple forms of delivery (injection, impregnated scaffolds, etc).

In this review, we clarify the historically-confusing nomenclature and cellular origins of calcifying EVs, and discuss the latest work on roles for EV accumulation in the initiation of microcalcification as well as their putative roles as mediators of intercellular communication in the vasculature. When aspects of this rapidly-progressing area are short on information from cardiovascular sources, we supplement with what is known from bone EV metabolism, and comment on the relevance of these findings to the vasculature. Finally, we discuss current gold-standard and forthcoming methods for the isolation, characterization, and study of EVs, and provide practical guidance for investigators interested in the study of EV-mediated vascular calcification.

## Release, Structure, and Composition of Calcifying Extracellular Vesicles

**Figure 2:**
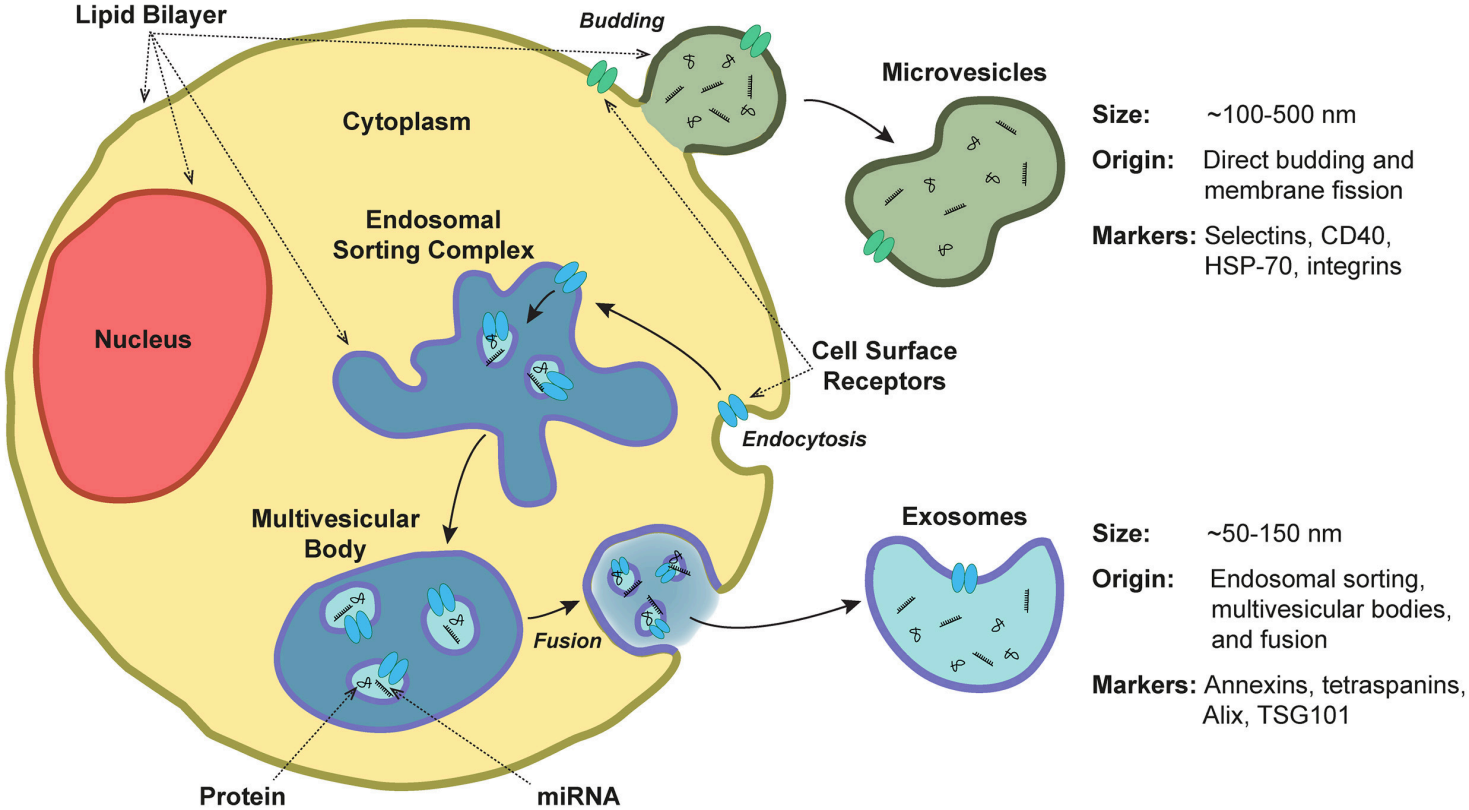
Extracellular vesicle release, structure, and composition. Extracellular vesicles are a heterogeneous population of secreted lipid bilayer-enclosed particles. Microvesicles are formed by direct budding and fission from the plasma membrane (~100–500 nm in diameter). In contrast, exosomes are released via the endosomal-sorting complex, where intraluminal vesicles formed by the inward budding of endosomal membrane are packaged within multivesicular bodies. These bodies then fuse with the plasma membrane to release their enclosed exosomes (~50–150 nm). Both exosomes and microvesicles contain miRNA and protein cargoes.

According to the guidelines of the International Society of Extracellular Vesicles (ISEV), the term “extracellular vesicles” collectively refers to secreted membrane-enclosed particles whose subsets have historically been referred to by field-dependent terms such as exosomes, ectosomes, microvesicles, microparticles, multivesicular bodies, matrix vesicles, and apoptotic bodies (25). Subset classification is based primarily on the route of biogenesis, as well as EV size, density, and protein markers. Cells release EVs through multiple processes (Figure 2): generally, exosomes refer to EVs released through the endosomal-sorting complex, where intraluminal vesicles formed by the inward budding of endosomal membrane are packaged within multivesicular bodies (MVBs). MVBs then fuse with the plasma membrane to release their enclosed exosomes (~50–150 nm in diameter) outside of the cell in a Rab GTPase-dependent manner [reviewed in (11, 26, 27)]. In contrast, microvesicles (or microparticles or “platelet dust”) are formed by direct budding and fission from the plasma membrane (~100–500 nm) (28). Other forms of secreted particles exist as well: during apoptosis, breakdown of the cytoskeleton induces bulges in the plasma membrane, which then separate as large apoptotic bodies (1,000–5,000 nm) (29). Even with the classification of these subsets, there is clearly substantial overlap between exosomes and microvesicles. Further complicating this scenario is the fact that these two populations are typically shed concurrently (30), a spectrum of heterogeneity exists within these subtypes (31), and there are currently no techniques that can identify the route of biogenesis beyond an estimate of EV size and subsequent correlation of size with known routes of release (see section Methods and Guidelines for Isolation of and Experimentation With Extracellular Vesicles).

Since 1967, the release of small vesicular bodies from cartilaginous epiphyseal plates has been implicated in the initiation of calcification (32–34). Epiphyseal chondrocytes, bone-derived osteoblasts, odontoblasts, etc. are known to release EVs that were originally termed matrix vesicles (MVs, not to be confused with microvesicles or multivesicular bodies), with diameters in the range of 100–400 nm (35, 36), and bud from specific locations on the plasma membrane [apical microvilli (36)]—indicating that at least a subset of MVs have a microvesicle-like origin (37). Macrophage-derived calcifying EVs are 30–300 nm in diameter (mean of ~50 nm), and originate as membranous protrusions (17). Electron microscopy demonstrates that MV-like vesicles are present in vascular calcifications of patients with chronic kidney disease (38) or atherosclerotic lesions (39). However, SMCs also release calcifying EVs that are typically on the order of 100–150 nm in diameter (19, 40), and recent work has shown that SMC-derived calcifying EVs originate from an exosomal pathway, are processed through multivesicular bodies, and are enriched in members of the Rab GTPase family (18). The structure of these calcifying EVs resembles that of exosomes, and inhibition of exosomal release in vascular SMCs inhibits their ability to mineralize (18). Importantly, MVs are also reported to be released from growth plate chondrocytes in such a way as to transit across intact plasma membranes (41), from epiphyseal cartilage as dense lysosomal bodies (42), and from osteoblasts as aggregates contained in a membrane-like sac that ruptures after release from the cell (43), indicating the presence of a MV subpopulation with exosomal origins. In short: (i) not all EVs are exosomes, (ii) all exosomes are EVs, (iii) osteoblasts/chondrocytes release mineralizing matrix vesicles that may be exosomes or microvesicles, (iv) macrophages release calcifying microvesicles, and (v) vascular SMCs release calcifying exosomes. Circulating vesicles from a myriad of other cell types (e.g., platelets, erythrocytes, leukocytes, MSCs, etc.) also likely contribute to the calcification of vascular plaques [reviewed in (44, 45)].

The intracellular origins of these calcifying EVs remain understudied, and are not always well-defined. Calcifying EVs can be produced by both exosomal and microvesicular modes of biogenesis, and vesicle release is clearly a highly dynamic process—cells can produce both forms of EVs (11). Importantly, many calcifying EV populations are described as having sizes that overlap with the 100–150 nm diameter range shared between prototypical exosomes and microvesicles. As noted above, recent work has clearly demonstrated that under high calcium/inorganic phosphate culture conditions (reminiscent of a CKD-like state), SMCs produce calcifying vesicles that are exosomal in origin (though their average diameter of ~140 nm places them on the high end of exosomal size) (18, 46). MVBs and exosomes have also been identified in the calcified arteries of dialysis patients, and appear to modulate mineralization in a fetuin-A- and sphingomyelin phosphodiesterase 3-dependent manner (18). In contrast, SMCs cultured under osteogenic conditions (rich in organic phosphate sources) produce larger calcifying EVs with a diameter of >150 nm. In 3D culture, these EVs mimic the collagen-dependent aggregation and calcifying behavior of microcalcifications found in mouse and human lipid/inflammation-rich atherosclerotic plaques. Consistent with this work, there is some evidence that macrophage-derived calcifying EVs have a microvesicular origin, as electron microscopy and time-lapse imaging has shown that they are released directly by budding from the plasma membrane (17). However, these calcifying macrophage-derived EVs have diameters of ~50 nm—more in line with that of exosomes. Regardless, these directly-budding EVs drive calcification through externalization of phosphatidylserine and the subsequent production of a phosphatidylserine-annexin V-S100A9 complex. The relative importance, contributions of, and mechanisms of actions for calcifying EVs derived from SMCs vs. macrophages remains to be determined. The limited experimental evidence available could indicate that microvesicular EVs may be the more dominant form of calcifying EVs produced in an inflammatory/atherosclerotic milieu, while exosomal calcifying EVs could play a larger role in CKD-associated calcification. In the future, rigorous identification and separation of putative EV sub-populations followed by proteomics-based phenotyping for biogenesis markers could enable a better understanding of the relative contributions of these differing routes of EV biogenesis to cardiovascular calcification. Specific inhibition of exosomal and, separately, microvesicle release pathways in model systems of disease would also ensure confirmation of the origins of the EVs of interest.

Despite their varied routes of biogenesis, EV subsets are all characterized by the presence of a membranous phospholipid bilayer. EV and cellular plasma membrane compositions differ: calcifying vesicle membranes are enriched in annexins and phosphatidylserines, which enhance their mineralizing potential (see section Biophysical Vesicle Aggregation and Mineralization) (47, 48). Internally and externally, exosomes and microvesicles carry protein, miRNA, and mRNA cargoes while apoptotic bodies are large enough to encapsulate nuclear fractions and entire organelle structures. As reviewed by van Niel et al. (11), exosomes/EVs are enriched in markers of their endosomal origins such as the members of the annexin family, tetraspanins (e.g., CD9, CD81, CD63), and proteins including Alix and TSG101 that are involved in exosomal biogenesis. They are also characterized by the presence of MHC complexes, lactadherin, RAB GTPases, and flotillin-1. Microvesicles, on the other hand, tend to be enriched in selectins (e.g., CD62), CD40, isoforms of heat shock protein-70, and various integrins. MVs are enriched in matrix metalloproteinases (MMPs, e.g., MMP-2,−3,−9,−13) that presumably assist with the remodeling of the extracellular matrix in order to expose charged sites on collagen fibers which are prone to initiation of mineral nucleation (49, 50).

In SMCs, there are large differences in cellular vs. vesicular miRNA populations, and a substantial number of miRNAs that are enriched in SMCs target genes involved in osteogenesis (51–54): miR-30,−125-b,−143,−145, and−155 modulate expression of proteins such as Smad1, Runx2, alkaline phosphatase (ALP, also known as tissue non-specific alkaline phosphatase or TNAP), and Osterix, resulting in altered MAPK signaling and calcium metabolism that drive vascular calcification (55). Next-generation sequencing reveals that osteoblast EVs have depleted levels of messenger RNAs (mRNAs) encoding proteins associated with basic homeostatic processes such as cytoskeletal function, cell survival, and apoptosis, and are enriched in mRNAs associated with protein translation, RNA processing, and stromal cell proliferation (56). There appears to be a disease-dependent localization of circulating miRNA: in plasma from healthy donors 90% of circulating miRNA is non-membrane-bound (57), whereas in patients with coronary artery disease the majority of plasma miRNAs are associated with EVs (58). In a CKD-mimicking environment (e.g., high-calcium, high-phosphate culture), EV loading with both inhibitors of calcification such as fetuin-A or matrix Gla protein (MGP) and drivers of mineral formation such as ALP is attenuated (18, 48). The nascent field of metabolomics has also begun to shed light on the small-molecule contents of EVs, and the potential for these cargoes to affect cellular function (59). Recent work has found that EVs can function as independent metabolic units with asparginase activity (60), with enrichment of a selected subset of cytosol-derived nucleotide and spermidine (autophagy-linked) pathway-derived metabolites (61).

## Biophysical Vesicle Aggregation and Mineralization

**Figure 3:**
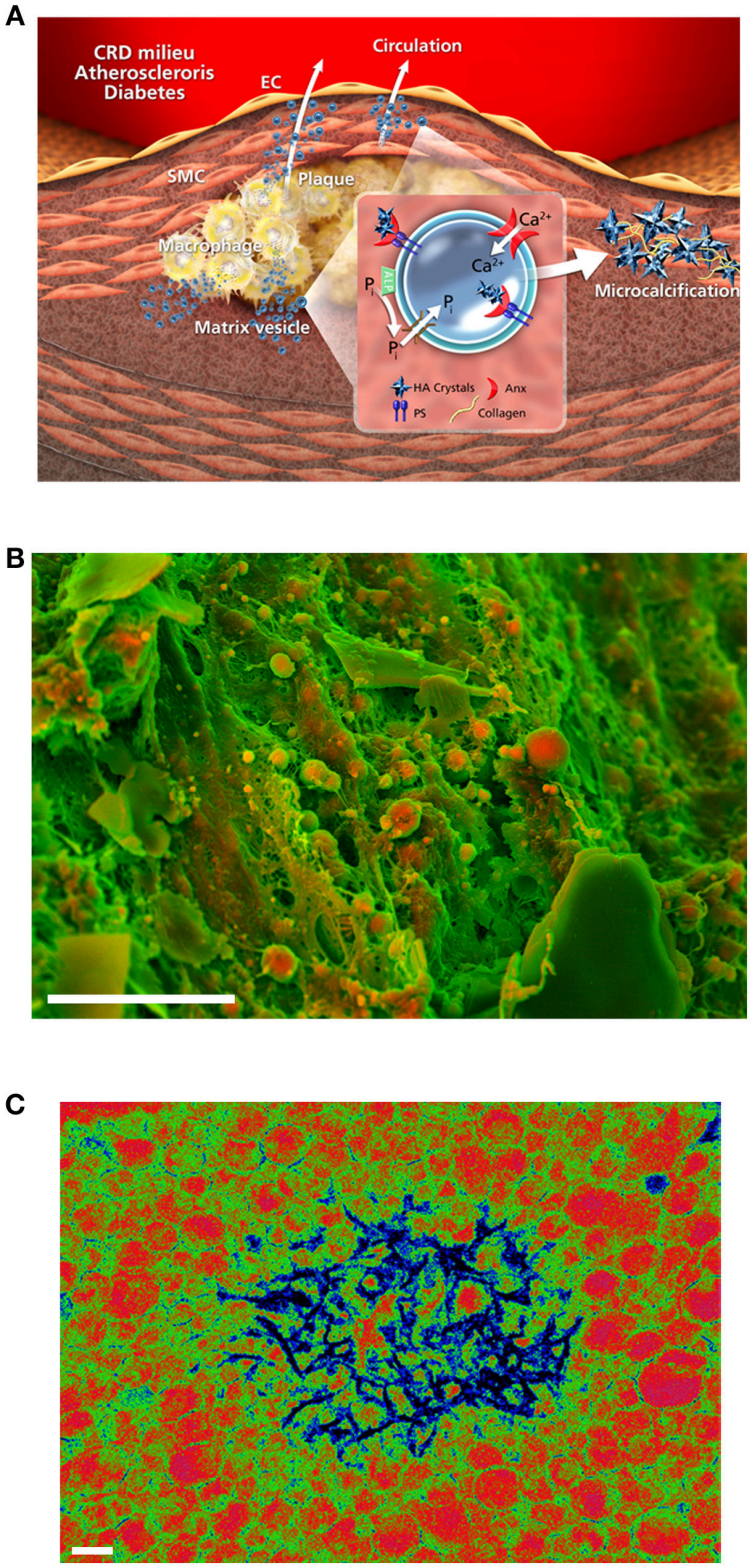
Accumulation and mineralization of calcifying EVs. **(A)** ALP produces inorganic phosphate (Pi) in the extravesicular space, and a mineral concentration gradient between the intra- and extra-vesicular spaces drives an influx of phosphate and calcium into EVs via suitable transporters. Hydroxyapatite nucleation is modulated by annexins and phosphatidylserines, leading to the production of calcified EVs and microcalcification; adapted from New and Aikawa (67). **(B)** Density-dependent color scanning electron microscopy (DDC-SEM) of calcified human atheroma (scale bar = 10 μm) reveals the accumulation of EVs as key building blocks of vascular calcification. **(C)** Pseudocolor transmission electron microscopy of macrophage-derived EVs reveals membrane-associated and intravesicular hydroxyapatite nucleation after calcium/phosphate treatment; adapted from New et al. (17) (scale bar = 200 nm).

Extracellular accumulation and aggregation of calcifying EVs occurs via several interrelated mechanisms, many of which involve EV interactions with the ECM. In both atherosclerotic plaque formation and fibrocalcific aortic valve disease, there is a robust fibrotic response and resultant accumulation of large amounts of disorganized collagen prior to the onset of calcification. Calcifying EVs contain high levels of collagen binding proteins [e.g., ALP, proteoglycan link proteins, hyaluronic-acid binding regions, annexins (62, 63)]. Exosomes also associate with other ECM components such as fibronectin by integrin binding (64), and expression of the collagen-binding receptor DDR-1 by SMCs modulates EV release and mineral deposition (21). As a counterpart to these collagen-binding sites, charged regions on the proteoglycans and collagen fibers of the ECM likely interact with charged EV membranes to promote accumulation via charge-charge interactions. Thus, in early-stage diseases characterized by tissue fibrosis, altered ECM turnover and degradation may expose charged structures that act as templates for EV binding and mineral nucleation (65). Local microenvironmental cues such as hypoxia may exacerbate EV trapping by collagenous ECM: circulating EV populations are enriched in an isoform of the collagen-crosslinking enzyme lysyl oxidase under hypoxic conditions (66). Using a novel 3D collagen hydrogel system paired with high-resolution optical and electron microscopy, Hutcheson and colleagues were able to directly observe the progressive aggregation of individual EVs to coalesce and then form microcalcifications (19). Notably, these microcalcifications are comparable to those of human atherosclerotic plaques (Figure 3) when detected by Fourier-transformed infrared spectroscopy (FTIR), indicating similar mineral maturation induced by the 3D model system. The most interesting findings of this study revolve around new evidence that EV aggregation kinetics may impact the stability of the fibrous cap. *In silico* modeling of human plaques has found that microcalcifications cause large increases in local tissue stresses, thereby potentiating plaque rupture (6, 7). In both 3D hydrogels and human plaques, there is an inverse relationship between collagen content and microcalcification size: as collagen degrades, EVs can accumulate, aggregate, nucleate hydroxyapatite, and form microcalcifications (19). Recent work from Hodroge and colleagues has identified specific regions on collagen fibers to which EVs bind: isoforms of 8-degree-polymerized oligogalacturonic acid mask the GFOGER sequence, inhibit vesicle-collagen binding, and impair the development of calcification (68).

How, then, does mineral first begin to nucleate and crystalize in EVs? While the specific location of nucleation remains unclear, under inflammatory or hyperlipidemic stimuli calcification is typically dependent on the increased expression and activity of ALP to free phosphate from a biological source (e.g., ATP) for nucleation. During cellular osteogenic transition, the multiligand sorting receptor sortilin regulates the release of EVs (69) and explicitly drives loading of activated ALP into calcifying EVs in a Rab11-dependent manner (70). Dimerization of this receptor appears necessary for the trafficking of sortilin, and thus ALP, into EVs (71). Notably, serum sortilin levels associate with increased risk of major adverse cerebrovascular and cardiovascular events, as well as with the severity of abdominal aortic calcification (72). In contrast, it appears likely that under hyperphosphatemic conditions (e.g., CKD or Monckeberg's syndrome), endocytosed phosphate binds with intracellular protein mediators of mineralization. Subsequently, exosomal EVs act to shuttle this mineral outside of the cell (18) in an ALP-independent manner. In bone, a precursor phase of amorphous calcium phosphate that acts as prenucleation clusters precedes hydroxyapatite crystallization (73). When compared with the parental cells (74), calcifying EVs are enriched in annexins, sphingomyelins, and phosphatidylserine (PS), a key mineral nucleation site on the EV membrane (75). Multiscale molecular dynamics simulations of calcium triphosphate Ca_2_(HPO_4_${)}_{3}^{2-}$ formation show that palmitoyloleoylphosphatidylserine has the greatest affinity for binding Ca^2+^ ions, with a ~4-fold greater contact with Ca^2+^ ions than palmitoyloleoylphosphatidylethanolamine (followed by palmitoyloleoylphosphatidylinositol, palmitoylsphingomyelin, and palmitoyloleoylphosphatidylcholine having progressively fewer calcium ion interactions) (76). In addition, coarse-grained simulations have found that ion:water concentrations of >0.26 are necessary for EV mineral nucleation to occur within reasonable time periods at physiological temperatures (76).

In addition to PS, there is ample indication that several members of the annexin family may also be direct drivers of EV mineralization, though it is notable that specific mechanisms of action for the regulation of calcification by this protein family remain unclear. The 14 annexins act as voltage-gated Ca^2+^ ion channels and/or as Ca^2+^-dependent anionic phospholipid binding proteins [reviewed in (77)], and are intimately involved in endo/exocytosis, membrane structure, and lipid raft organization [reviewed in (78)]. Typically, EVs are enriched in annexins A2, A5, and A6, and annexin-enriched EVs derived from osteoblasts drive mineralization of cultured mesenchymal stem cells (MSCs) (79). Calcium and phosphate stimulation of macrophages induces externalization of PS and a resultant interaction of PS, annexin A5, and the calcium-binding protein S100A9 that leads to hydroxyapatite nucleation and which likely contributes to plaque calcification in CKD (17). Annexin A6 is preferentially increased in exosomes derived from calcifying vascular SMCs, where overexpression/knockdown studies demonstrate that it appears to function as an active mediator of ECM mineralization (18, 48). In calcified aortic valve leaflets, annexin A6 co-localizes with EV and is elevated in rat VIC-derived EVs under high calcium/phosphate conditions (80). Such high-phosphate conditions also increase shedding of calcifying EVs from both odontoblasts (81) and vascular SMCs (82) while driving the incorporation of annexin family members into EVs derived from the latter. Annexin A2 also prevents loading of the mineralization inhibitor fetuin-A into vascular SMC-derived EVs by directly binding to and inducing its endocytosis (63). Indeed, in both vascular SMC and osteoblast-derived EVs, increased annexin A2 levels result in a concomitant activation of EV ALP and calcification potential (63, 83).

Beyond annexins and phosphatidylserines, there is a large body of evidence that homeostatic loading of EVs with inhibitors of calcification such as fetuin-A and MGP (16) is disrupted in calcifying EVs (48), leading to a loss of inhibition of mineralization. Recent work by Viegas and colleagues found that serum from patients with advanced CKD contains EVs with reduced levels of fetuin-A, and Gla-rich protein (GRP) (84). Notably, circulating GRP-rich EVs appear to be produced by monocyte/macrophage populations and have additional anti-inflammatory functionality through the down-regulation of proinflammatory cytokine expression (85). Other recent proteomics studies have identified roles for the deposition of EV-derived prothrombin in modulating calcific burden and thrombogenesis in the vasculature (46). These EVs drive vascular SMC calcification via inflammatory induction of an osteochondrogenic phenotype, and this study determined that production of calcium phosphate crystals can be reduced specifically by the addition of γ-carboxylated GRP *in vitro* (84). In concert, EV levels of proteins that drive intracellular Ca2^+^ concentration (e.g., NADPH oxidase) and modulate oxidative stress (e.g., SOD2) are enriched (86). Proteomics of an ultracentrifugation-isolated calcifying EV population derived from human coronary artery SMCs demonstrates significant enrichment in proteins associated with glycosaminoglycan binding, calcium ion binding, ECM binding, and nitric oxide synthase regulation, among others (40). Most recently, the importance of cellular senescence to atherosclerotic calcification has come to light—senescent macrophages accumulate in the subendothelial space at disease onset, SMCs in advanced atherosclerotic plaques are highly senescent, and the deletion of senescent cells in mouse models of atherosclerosis and vascular calcification drives substantial lesion regression (87, 88). EVs isolated from the plasma of elderly donors or from cultures of senescent endothelial cells promote the calcification of human aortic SMCs; as subjects age, these EVs are more prevalent and contain greater amounts of calcium, annexin A2, annexin A6, and bone morphogenic protein-2 (BMP2) (89).

## Roles in Intercellular Communication

**Figure 4:**
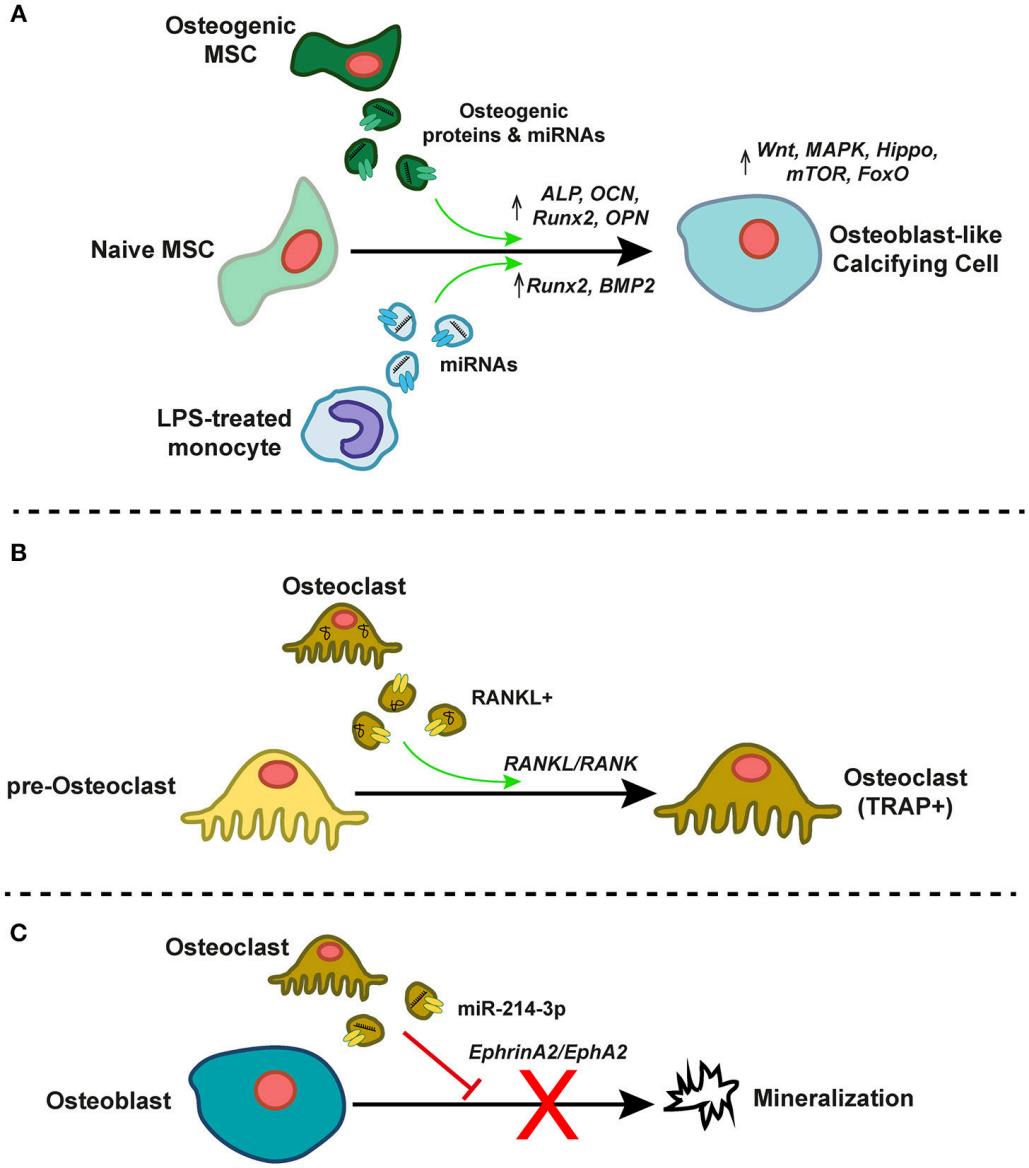
EVs as mediators of intercellular communication in the calcifying milieu. **(A)** EVs from osteogenic mesenchymal stem cells (MSCs) or lipopolysaccharide (LPS)-treated inflammatory monocytes contain pro-osteogenic proteins and miRNAs that induce pro-osteogenic gene expression and signaling programs in recipient naïve MSCs, thereby driving differentiation toward an osteoblast-like calcifying cell type. **(B)** TRAP- pre-osteoclasts differentiate to mature TRAP+ osteoclasts after treatment with RANKL-containing EVs derived from mature osteoclasts. This EV-associated RANKL induces RANK signaling in recipient pre-osteoclasts. **(C)** Osteoclast-derived EVs enriched in miR-214-3p inhibit mineralization by mature osteoblasts, perhaps via ephrinA2/EphA2 signaling.

As noted above, EVs are selectively enriched in specific miRNA, mRNA, and protein cargoes, and are utilized by cells as a means of local or long-distance intercellular paracrine/endocrine communication [Figure 4; (90)]. Functionally, the EV lipid bilayer serves to protect miRNAs/mRNAs and proteins from degradation by extracellular ribonucleases and proteases, respectively (91, 92)—thereby dramatically increasing the plasma/tissue half-life of these compounds in comparison to their un-packaged forms. As an example, preserved phosphoproteins have been recovered from exosomal samples even after 5 years of cryopreservation (92). While a not inconsequential portion of extracellular RNA is associated with Argonaute 2 protein complexes and not vesicles, the composition of these two pools is significantly different (57) and both differ from that of their donor cells (58). It is therefore likely that cells preferentially and actively select miRNAs for packaging into EVs. Substantially less is known about whether or how EV protein cargoes are processed or packaged. The advent of inexpensive, higher-throughput proteomics and low-input peptide isolation techniques (93) may help to tackle the question of whether protein pools are similarly regulated.

Calcific and inflammatory burdens in both the arteries and aortic valve correlate with paradoxical osteoporotic bone remodeling (94), and so the well-described phenomena of EV-mediated control of osteoblasts- and/or osteoclastogenesis is of particular relevance to vascular calcification. EVs released by actively mineralizing pre-osteoblastic cells promote differentiation of recipient bone marrow stromal cells (BMSCs) to an osteogenic phenotype (95), and the proteome of these vesicles is enriched in pathways associated with exosome biogenesis, formation, uptake, and osteogenesis (96). This EV-induced osteogenic differentiation appears to be sensitive to extracellular magnesium levels, likely due to induction of autophagic processes (97). In concert, miRNA profiles in recipient pre-osteoblastic cells change significantly due to exosomal miRNA transfer, and result in activation of Wnt/β-catenin signaling via inhibition of Axin1 (95). Furthermore, treatment of human fetal pre-osteoblasts with EVs isolated from BMSCs induces osteogenic gene expression (ALP, OCN, Runx2, OPN), osteoblastic differentiation, and mineralization. These BMSC-derived EVs are enriched in the pro-osteogenic miRs−27a,−196a, and−206, and pharmacological abolishment of EV miR-196a partially inhibits EV-induced osteogenic gene expression (98). *In vivo*, hydrogel-based delivery of BMSC-derived EVs to rat critical-sized calvarial bone defects promotes bone regeneration. Interestingly, these EVs were derived from BMSCs under normal culture conditions, thus implying that even basal state EVs from these cell types have the ability to induce an osteogenic phenotypic switch. There is also evidence that EV-induced osteoblastogenesis is sensitive to the temporal stage of osteogenesis of the EV “donor” cell population. Vesicles collected from human MSCs at both early and late stages of osteogenic differentiation can induce ALP expression in naïve homotypic cells, but only those derived from late-stage osteogenic MSCs produce mineralization of the ECM in recipient cell cultures. This effect is mediated, at least in part, by significant temporal alterations in 16 EV miRNAs whose functions are associated with the modulation of the Wnt, MAPK, Hippo, mTOR, and FoxO signaling pathways—all of which are involved in regulation of osteogenesis (99). The pro-osteoblastogenic effect of EVs is not confined to those derived from osteoblast-like cells. An inflammatory stimulus of miRNA-containing EVs from LPS-treated monocytes drives increases in osteogenic gene programs (e.g., Runx2, BMP2) in human MSCs within 72 h of EVs being taken up (100, 101).

Understanding osteoblast-osteoclast interplay and EV-induced osteoclastogenesis in the cardiovascular system is paramount. Vesicles shed from osteoblasts contain the receptor activator of nuclear factor kappa-β ligand (RANKL), and LAMP1-positive EVs shed by both osteoblasts and osteoclasts also contain tartrate-resistant acid phosphatase (TRAP) and osteoprotegerin (OPG) (102). When administered to RAW264.7 osteoclast precursors, these EVs deliver RANKL, causing stimulation of RANKL-RANK signaling, and differentiation of the recipient osteoclast precursors to become TRAP-positive osteoclasts (103). In contrast, while EVs from murine pre-osteoclasts drive mouse marrow hematopoietic precursors to differentiate to TRAP-positive osteoclasts, those from mature osteoclasts do not induce osteoclastogenesis in recipient cultures, but rather inhibited it (104). Mature osteoclast-derived EVs are enriched in RANK, and subsequent depletion of RANK in these EVs restored their ability to induce osteoclast differentiation. RANK-positive EVs may competitively bind to RANKL (in a similar manner to RANKL/OPG binding), thus inhibiting osteoclastogenesis. Alternatively, mature osteoclastic EVs may deliver other regulatory cargoes (104). Osteoclasts may also regulate osteoblastic bone formation via EVs (105, 106). Levels of exosomal miR-214-3p are highly associated with inhibition of bone formation in elderly women with fractures (107), and overexpression of miR-214-3p in osteoclasts inhibits osteoblast-mediated mineralization in co-culture (107). This phenomenon may be mediated by ephrinA2/EphA2 signaling (106). Together, these findings indicate the presence of a complex, EV-dependent paracrine regulatory system of osteoblast/osteoclastogenesis [reviewed in (108)]. Despite this multitude of studies, exact mechanisms and the specific miRNAs/proteins responsible for this cross-talk remain elusive, as does confirmatory evidence of *in vivo* EV crosstalk in vascular calcification.

Endothelial-derived EVs from both patients with acute coronary syndrome and senescent endothelial cell (EC) EC cultures can induce early senescence in receptor ECs (the former of which occurs in a shear-sensitive manner at low atheroprone shear stresses) and are associated with increased levels of receptor cell oxidative stress, MAPK and Akt signaling, and upregulation of p53 (109). These findings demonstrate that EVs released from senescent cells can also drive senescence of neighboring cells in a paracrine manner, and EV levels/composition may serve as important diagnostic biomarkers of vascular calcification. For example, urinary exosomal fetuin-A levels are a strong predictive biomarker of acute renal injury (110). Other studies have shown that SMC phenotypes in the vessel wall are regulated by EC-derived EVs that are selectively loaded with miR-126,−143, and−145 (111, 112).

There is also evidence of EV-driven cell-cell transfer of mRNA: in co-culture, next generation sequencing shows that a multitude of mRNAs are transferred through EVs between differing cell types. The function of these mRNAs ranges from transcriptional regulation to protein coding, and their biological roles are associated with ECM regulation, cell adhesion, glycoproteins, and signal peptides (113). One long-time mystery in valvular biology has been the question of how valvular endothelial cell (VEC)/VIC paracrine signaling occurs—how do VEC-detected changes in shear stress, stretch, circulating growth factor, and/or lipid levels modulate VIC function, and vice-versa (114). In VIC/VEC co-cultures, VIC-derived EVs are taken up into the perinuclear space by VECs (115) through the endosomal pathway, and EV transfer of miRNA, mRNA, and protein may therefore be a key mode of VEC/VIC communication.

## Therapeutic Potential

Along with their clear implication in the pathogenesis of cardiovascular calcification, EVs may also hold the key to successfully treating these pathologies in the clinic. Recently, a number of studies have begun to explore the notion that EVs could themselves have a unique ability to act as therapeutic agents [reviewed in (116)]. Indeed, the fact that they are so centrally positioned in the progression of mineralization means that delivery of therapeutic forms of EVs or disruption of the contents, synthesis, release, or extracellular accumulation of endogenous pro-calcifying EV populations could efficaciously prevent or regress calcification. MSC-derived exosomes have shown positive results in a number of disease models, such as reduction of myocardial infarction size and improvements to post-MI inflammation (117, 118), resolution of pulmonary hypertension (119), amelioration of kidney fibrosis (120), and restoration of neurovascular function and plasticity (121). Importantly, EVs derived from immunocompetent MSC populations may themselves also be immunocompetent—thus providing a non-means of delivering complex biological cargoes [reviewed in (122)]. One popular approach that can maintain tissue homeostasis or reverse cardiovascular pathogenesis is to deliver EVs collected from the conditioned media of cultured “producer” cells [reviewed in (123)]. Cargo loading generally occurs either during biogenesis (by overexpression of cargo in the producer cells and relying on increased intracellular levels to enrich EV levels), or by post-biogenesis, by collecting EVs then subsequently loading them by transfection or hydrophobic tagging. miRNAs and siRNAs have been successfully loaded into EVs by electroporation for nearly a decade (124, 125). EVs can deliver miRNAs to difficult-to-transfect cells such as MH-S lines and bone marrow-derived macrophages (126), and more contemporary approaches that leverage conjugation of RNA with hydrophobic cholesterols have increased loading efficiencies to nearly 50% (127). While RNA cargoes have been most frequently employed, protein or small molecule loading is frequently a more attractive proposition (obviates the need for transcription/translation in treated cells, along with the advantage of inherently higher cargo stability). By tagging with a WW domain, protein of interest can be forced to interact with ubiquitination-promoting Ndfip1, that then drives loading of the target protein into exosomes (128). As an alternative light-inducible and reversible approach (i.e., spatially/temporally controlled loading and controlled release), cargo proteins can be tagged with the photoreceptor CRY2 while CD9 is fused with a truncated CRY-interacting domain. Upon exposure to blue light, these domains interact and the complex is loaded into EVs (129). In addition, small molecules such as doxorubicin and porphyrins have been loaded into EVs by electroporation, saponin treatment, and extrusion (130, 131), though encapsulation efficiency is often <20%. Another option is to use fusogenic liposomes and a packaging cell line: the small molecule of interest is incorporated into liposomes, which efficiently fuse with the cellular plasma membrane, delivering the small molecule into the packaging cells. EVs produced by those cells (and naturally enriched in the molecule of interest due to mass action) can then be collected (132).

Though the advantages of active EV loading are clear, they do come with additional regulatory requirements centered on demonstrations of purity. As an alternative, vesicular secretome fractions (VSFs) or naïve, unmanipulated EVs derived from cell types relevant to the pathology of interest can be employed. EVs from clonal cardiac stem cells mitigate ventricular damage in animal models of acute myocardial infarction (133). Oxidative stress (frequently coincident with vascular calcification) causes uncoupling of endothelial nitric oxide synthase (eNOS) and endothelial dysfunction. EVs derived from cultured human umbilican vein endothelial cells (HUVECs) carry functional eNOS and protect the vasculature against oxidative stress by Akt-mediated upregulation of superoxide dismutase under *ex vivo* culture conditions. Despite these promising initial efforts having become a key focus of the drug discovery field, the impact of exosomal therapeutics on vascular calcification remains unstudied. A degree of care will be required when such studies are undertaken, as MSC-derived EVs can—under the proper conditions—robustly drive osteogenic differentiation, endochondral ossification, and bone regeneration (95, 98, 134). Besides using exosomes as a delivery vehicle, the contents of calcifying EVs are proving to be an important source of drug targets in and of themselves. Treatment with the inhibitor of acid sphingomyelinase imipramine reduces calcifying EV release from osteoblasts (135), and the Ca^2+^ channel blocker verapamil reduces SMC calcifying EV biogenesis, vesicle ALP activity, and ECM mineralization, while also impairing atheroma formation in the rat aorta (136). Dimerization of the aforementioned sortilin protein also appears to regulate its packaging in EVs—inhibition of sortilin homodimer formation is therefore another promising EV-associated therapeutic strategy (71). EVs thus offer the promise of a biological, acellular, targeted, and immunocompetent means of delivering complex therapeutic cocktails to calcifying cardiovascular tissue (24).

## Methods and Guidelines for Isolation of and Experimentation with Extracellular Vesicles

There are several important methodological considerations when performing experiments involving exosomes or EVs. As this field has matured, a number of vesicle isolation techniques have become popular (Table 1), however EV yield, integrity, biodistribution, clearance, and breakdown are all affected by the choice of isolation method (137). Ultracentrifugation is one of the most simple, widely-available, and cost-effective strategies for vesicle collection, though it will promote vesicle clumping and may cause soluble factors and ECM-rich protein aggregates to pellet along with EVs themselves (138). Recovery is typically variable (10–80%), and is capable of concentrating a sample ~8x (139). Fortunately, calcifying EVs have an increased density over other EVs (due perhaps to elevated mineral content) and pellet more quickly under ultracentrifugation (40). Alternative methods include density gradient isolation/flotation, where ultracentrifugation is paired with sucrose or ioxidanol gradients to obtain fractions enriched in EVs based on mass density and can obtain EV recovery in the range of 5–50% (140). Care must be taken when using sucrose, as there is evidence that non-isosmotic sucrose concentrations cause leaching of vesicle cargoes during isolation [summarized in (141)]. Size exclusion chromatography can be performed on a single column, removes 99% of soluble plasma proteins and does not induce EV aggregation, though it is prone to co-isolation of viruses, protein aggregates, and very large proteins (142–144). Recovery rates are typically 30–90% of EVs (145). Ultrafiltration's ability to concentrate from soluble components EVs is currently unmatched, with an ability to do so up to 250x with nearly an 80% recovery (146). To isolate specific subpopulations of EVs, immunocapture assays using immobilized monoclonal antibodies can be utilized (147). Non-EV proteins are often recovered in these assays, and care must be taken to account for cross-reactivity and non-specific binding (31, 148). Recently, commercially-available kit-based precipitation approaches have become popular due to their simplicity and ease of use. These kits leverage the volume-excluding properties of polyethylene glycol polymers (149) to reduce EV solubility (150). Recovery of ~90% and a 50x concentration performance can be expected by these kits (151). Caution is necessary when attempting to quantify precipitation-isolated EVs by nanoparticle tracking analysis, as the polymer components of the precipitation buffer appear to frequently be detected as false-positive EV particles. Besides these standard approaches, a number of innovative techniques are also under development. By using ultrasonic standing waves, acoustic separation can exert differential force on exosomes in a scalable manner (152). Flow field fractionation exploits perpendicular flow fields to separate exosomes by their size-based diffusivity (153). Lastly, microfabricated micropillar-based nano-traps can be tuned to capture EVs in a particular size range of interest, while washing out cellular debris, other EV size fractions, and cellular debris (154). It is important to note that each of the methods described above has its own unique sets of tradeoffs (on ease of use, availability, cost, recovery, concentration, etc.), none is perfect, and there exists no consensus on a single gold-standard isolation approach. Instead, investigators must make an effort to comprehensively report the method that was selected, and to rigorously assess and describe the phenotype of their isolated EV population (e.g., purity, concentration, size range, morphology).

**Table 1:** Methods of extracellular vesicle isolation.

**Isolation method**	**Working principle**	**Volume reduction (fold)**	**EV recovery (%)**	**Notes**
Ultra-centrifugation	Size separation: large EVs collect earlier & at lower g forces	8	10–80	Cost-effective; causes vesicle clumping; pellets soluble factors & ECM along with EVs
Density gradient centrifugation	Separation by density in sucrose/iodixanol gradients	1	5–50	Non-isosmotic sucrose concentrations cause leaching of EV cargoes
Size exclusion chromatography	Column-based size separation	0.2	30–90	Effective separation of plasma proteins; prone to co-isolation of large proteins/aggregates
Ultrafiltration	Pressure-mediated by size/solubility	250	80	EV deformation possible; co-isolation of EV-particle aggregates
Immunocapture	Immobilized antibodies against EV-specific ligands	5	N/A	Cross-reactivity & non-specific binding: recovery of non-EV proteins
Precipitation	PEG polymers exclude water volume	50	90	Inexpensive; concerns re: compatibility with NTA

Though researchers have several options available in order to characterize EV populations of interest, electron microscopy (EM) is the gold standard for exosome imaging. The resolution of this technique is ~1–5 nm (139), allows for multiplexed immunogold labeling to phenotype EVs, and can reveal EV population size distributions, presence of aggregation, and inclusion of debris contaminants. EM is not, however, able to measure EV concentrations. The recent advent of density-dependent scanning electron microscopy (SEM) allows for *in-situ* imaging of calcifying EVs and assessment of their size, density, and biophysical accumulation in calcified tissues, hydrogels, or matrices (19). *In situ* imaging of vesicles in concert with (immuno)fluorescence/confocal light-based approaches is now feasible with the advent of super-resolution microscopy (e.g., SIM, TIRF, PALM, or STORM), which overcomes the traditional resolution limitations that diffraction places on light microscopy (155). These imaging techniques enable resolutions as low as 20 nm, sufficient to visualize individual exosomes within a physiological ECM (19).

Though typically used to count, phenotype, and/or separate cells, flow cytometry can also be applied to EVs—with the essential caveat that because EVs are orders of magnitude smaller than cells, light scattering from these exosomes may be close to, or below, the background noise detected on current-generation flow cytometers (156). Combinatorial counting that leverages tags, membrane dyes, and/or antibodies against vesicle markers (e.g., annexins) can improve this performance (157). Instrument settings must be tuned for EV analysis, and one must remember that for the purposes of calibration, the size of EVs cannot be related directly to the size of calibrator polystyrene/silica beads due to differences in refractive index (158), though models do exist that attempt to account for these elements (159). By passing EVs through a pore and measuring a resultant change in electrical impedance, resistive pulse sensing (RPS) can quantify exosome size and electrophoretic mobility (160), though it cannot differentiate between EVs and contaminating particles (139) and pores can be prone to clogging if samples are not pre-filtered and/or have already undergone size exclusion chromatography (161). One of the most commonly used and cost-effective means of measuring EV size distributions and concentrations is nanoparticle tracking analysis (NTA), which optically tracks vesicle Brownian motion (162). Many NTA instruments also incorporate measurement of membrane charge state, or zeta potential. A sufficiently-large number of particles (typically >4,000) must be measured to ensure the statistically-relevant accuracy of the size distribution, and reference particles should be utilized for concentration calibration (139).

Once EVs have been isolated, quantified, and characterized, their contents can then be measured. Next-generation sequencing has been used to examine EV RNA populations derived from a number of different cell types/microenvironmental milieus (163, 164), and rigorous guidelines have been developed for the use of NGS techniques on exosomes (165). qPCR and microarray techniques can be used for EV RNA analysis, but both lack an ability to identify novel sequences and do not perform as well as NGS when quantifying transcript numbers (139). Both miRNA and mRNA expression profiles differ based on isolation technique [e.g., precipitation vs. ultracentrifugation (166)], with iodixanol density gradients having the best purity performance (167). At the next stop on the central dogma, EV protein levels are frequently assessed by Western blotting or ELISA. However, the advent of proteomic approaches to quantify thousands of proteins has revolutionized this approach—nearly 10,000 vesicle-associated proteins have been annotated (168, 169). While care must be taken to remove or account for co-isolating non-EV proteins, recent advantages in density-dependent ultracentrifugation-based isolation of SMC-derived calcifying EV populations enabled LC-MS/MS identification and quantification of over 400 EV proteins, with subsequent pathway analysis finding calcification-associated functionalities (40). Lastly, EVs also carry cellular metabolites that may provide insight into the biochemical status of their originating cells, and mass spectrometry-based metabolomics techniques to assess the EV metabolome have recently begun to be presented (60, 61, 170, 171). Importantly, label-free and non-targeted metabolite identification is a massively more complex undertaking than global proteomics, and metabolite libraries must be carefully and rigorously employed to confirm target IDs (172).

One additional area of caution should be noted when working with EVs: when cell types relevant to vascular calcification are cultured (e.g., endothelial, SMCs, VICs, monocyte/macrophage, osteoblast, osteoclasts, etc.), the culture media is typically supplemented with ~1–10% fetal bovine serum (FBS) in order to provide essential growth factors and adsorbed ECM components that aid in cell attachment. Importantly, even commercially-available lots of FBS are replete with EVs and exosomes (often somewhere on the order of ~10e^10^ exosomes per ml). Besides acting as a contaminating source of extracellular RNA or protein when co-isolated with endogenously-produced EVs (173), this undefined and highly variable component of the culture conditions can have an outsized effect on cell behavior (174, 175). Various strategies are used to account for this: exosome-depleted serum can be purchased, though (i) it is costly, (ii) there is currently no exosome-depleted human serum on the market, (iii) producing it in-lab typically requires access to an ultracentrifuge, (iv) such serum is only exosome-depleted (of ≥90% of endogenous serum exosomes), not exosome-free, and (v) there is evidence that exosome-depleted serum has a reduced capacity to support cell growth (176). Alternatively, cells can be cultured in normal concentrations of FBS to support growth, attachment, and phenotype, then switched low concentrations of FBS (e.g., incomplete media or 0.1% FBS) for the final portion of an experiment. This solution is not ideal either; as differing FBS levels alter endogenous cellular exosome production, release, and contents. Furthermore, if EV-ECM interactions are relevant to the study aims or the milieu being modeled [e.g., experiments performed on 3D hydrogels, *ex vivo* tissue culture, or matrix-bound vesicles (63)], then a short-term switch to incomplete media will have a limited capacity to flush/remove FBS-derived EVs that have built up in the ECM throughout the remainder of the experimental timeline. One solution: in proteomics experiments, the species of detected/sequenced proteins can be determined with a reasonable degree of accuracy (177). If the cells being studied are non-bovine, then the entire extracellular EV proteome can be isolated, detected, and sequenced at once, followed by filtering out all proteins identified as bovine in origin. Unfortunately, this is much more difficult with miRNAs due to their high degree of conservation across species (178). Regardless, great care should be taken to minimize and account for any exogenous vesicle contamination from culture media.

## Conclusions and Future Directions

The discovery of calcifying EVs has led to a growing appreciation for the key role that these bodies play as both active and passive constituents of vascular calcification. One appropriate analogy may be that of masonry: by inherently regulating their own aggregation and mineralization, EVs act as both the bricks and the mortar of microcalcifications. Importantly, they also actively control the work of the bricklayers themselves (e.g., osteoblasts, osteoclasts). In short, EVs seem to be intimately connected to all stages of the pathogenesis of vascular calcification. As this burgeoning field matures, a number of emerging questions remain unanswered: Can we identify specific roles for EV microRNAs in preventing or promoting vascular calcification? *In vivo*, how does osteoblast/osteoclast vesicle cross-talk *in vivo* modulate mineralization of vascular tissue in both homeostasis and disease? By what specific mechanisms do annexins mediate calcifying vesicle aggregation and mineralization, and how do annexins interact with sphingomyelin and phosphatidylserine to promote nucleation? Can EVs be therapeutically administered locally/systemically without off-target effects (e.g., unintended promotion of vascular osteogenesis and/or skeletal osteoporosis). In the future, the benefits of better understanding the mechanistic basis of vascular EV calcification may be two-fold: first, robust study of calcifying EVs could yield a novel set of anti-calcification drug targets for small molecule high-throughput screening that are largely distinct from existing (intra)cellular targets due to the selective loading of EV cargoes. Second, purposeful introduction of exogenous engineered EVs to the calcifying vascular milieu might be a viable and revolutionary therapeutic delivery strategy, whereby inhibitors of osteogenesis and mineralization or inducers of osteoclast differentiation could be efficiently conveyed into atherosclerotic plaques or fibrocalcific valve leaflets. Indeed, such an approach could circumvent many of the issues associated with intracellular delivery of small-molecules and/or nucleic acids. In the long run, it may even be feasible to develop artificially-functionalized EVs that leverage the physicochemical mechanisms of mineral formation in order to locally arrest mineral nucleation, prevent further aggregation of endogenous calcifying EVs, and even stabilize vulnerable atheromata.

## Author Contributions

MB performed an extensive literature review, drafted the manuscript, prepared figures and tables. EA proposed the subject of the review, and critically revised and edited the manuscript.

### Conflict of Interest Statement

The authors declare that the research was conducted in the absence of any commercial or financial relationships that could be construed as a potential conflict of interest.

## Abbreviations

ALP: alkaline phosphatase
BMSC: bone marrow stromal cell
CKD: Chronic kidney disease
ECM: extracellular matrix
EC: endothelial cell
EV: extracellular vesicle
LPS: lipopolysaccharide
MGP: matrix Gla protein
MSC: mesenchymal stem cell
MV: matrix vesicle
MVB: multivesicular bodies
OPG: osteoprotegerin
RANK: receptor activator of nuclear factor kappa-β
RANKL: receptor activator of nuclear factor kappa-β ligand
SMC: smooth muscle cell
TNAP: tissue non-specific alkaline phosphatase
TRAP: tartrate-resistant acid phosphatase
VEC: valvular endothelial cell
VIC: valvular interstitial cell.
